# ICELLNET v2: a versatile method for cell–cell communication analysis from human transcriptomic data

**DOI:** 10.1093/bioinformatics/btae089

**Published:** 2024-03-15

**Authors:** Lucile Massenet-Regad, Vassili Soumelis

**Affiliations:** Université Paris Cité, INSERM U976 HIPI, Paris, F-75010, France; Université Paris-Saclay, Saint Aubin, F-91190, France; Université Paris Cité, INSERM U976 HIPI, Paris, F-75010, France; Department of Immunology-Histocompatibility, Saint-Louis Hospital, AP-HP.Nord, Université Paris Cité, Paris 75010, France; Owkin France, Paris 75010, France

## Abstract

**Summary:**

Several methods have been developed in the past years to infer cell–cell communication networks from transcriptomic data based on ligand and receptor expression. Among them, ICELLNET is one of the few approaches to consider the multiple subunits of ligands and receptors complexes to infer and quantify cell communication. In here, we present a major update of ICELLNET. As compared to its original implementation, we (i) drastically expanded the ICELLNET ligand-receptor database from 380 to 1669 biologically curated interactions, (ii) integrated important families of communication molecules involved in immune crosstalk, cell adhesion, and Wnt pathway, (iii) optimized ICELLNET framework for single-cell RNA sequencing data analyses, (iv) provided new visualizations of cell–cell communication results to facilitate prioritization and biological interpretation. This update will broaden the use of ICELLNET by the scientific community in different biological fields.

**Availability and implementation:**

ICELLNET package is implemented in R. Source code, documentation and tutorials are available on GitHub (https://github.com/soumelis-lab/ICELLNET).

## 1 Introduction

The coordination of multicellular biological processes, such as organ development, homeostasis, or activation of the immune system, highly relies on cell–cell communication. Dysfunctions in cell–cell communication mechanisms can lead to pathologies. The massive technological developments in large-scale transcriptomics, in particular with single-cell RNA sequencing (scRNAseq) data, have enabled the reconstruction of cell–cell communication networks between several cell types in human tissues or pathological contexts (Armingol *et al.* 2021). Despite having their own specificities, underlying methodologies mostly rely on the expression of ligand and receptor genes to evaluate cell–cell communication (Efremova *et al.* 2020, Jin *et al.* 2021, Noël *et al.* 2021), and more rarely on downstream target genes or signaling pathways (Browaeys *et al.* 2020).

We have previously developed ICELLNET, which computes communication scores between cells from their transcriptomic profiles relying on a manually curated database of ligand–receptor interactions (Noël *et al.* 2021). Interactions included in ICELLNET are demonstrated experimentally in human systems and encompass the multiple subunits of protein complexes. ICELLNET has been applied to wide range of transcriptomic data and revealed interesting communication channels in the tumor microenvironment (Shaashua *et al.* 2022, Hoffmann *et al.* 2022, Massenet-Regad *et al.* 2023), as well as immune crosstalk in the blood of SARS-CoV-2 (Saichi *et al.* 2021) and chronic hepatitis B virus infected patients (Zhong *et al.* 2022). Recent benchmarking studies (Liu *et al.* 2022, Luo *et al.* 2023) identified ICELLNET among the most performing methods to accurately reconstruct cell–cell communication networks. However, the original ICELLNET database incorporated a relatively low number of interactions (Dimitrov *et al.* 2022).

The content of ligand–receptor interactions database has been shown to considerably affect cell-cell communication analysis results (Dimitrov *et al.* 2022, Liu *et al.* 2022). In addition, some technical challenges remain: (i) Intrinsic limitations of scRNAseq data, such as dropout and technical noise, can lead to false positive and false negative results; (ii) cell–cell communication analyses generate extensive results that are complex to handle and interpret. In this work, we addressed those challenges to optimize ICELLNET and extend its performance and application field.

## 2 Major increase of ICELLNET ligand–receptor interaction database

**Figure 1.**
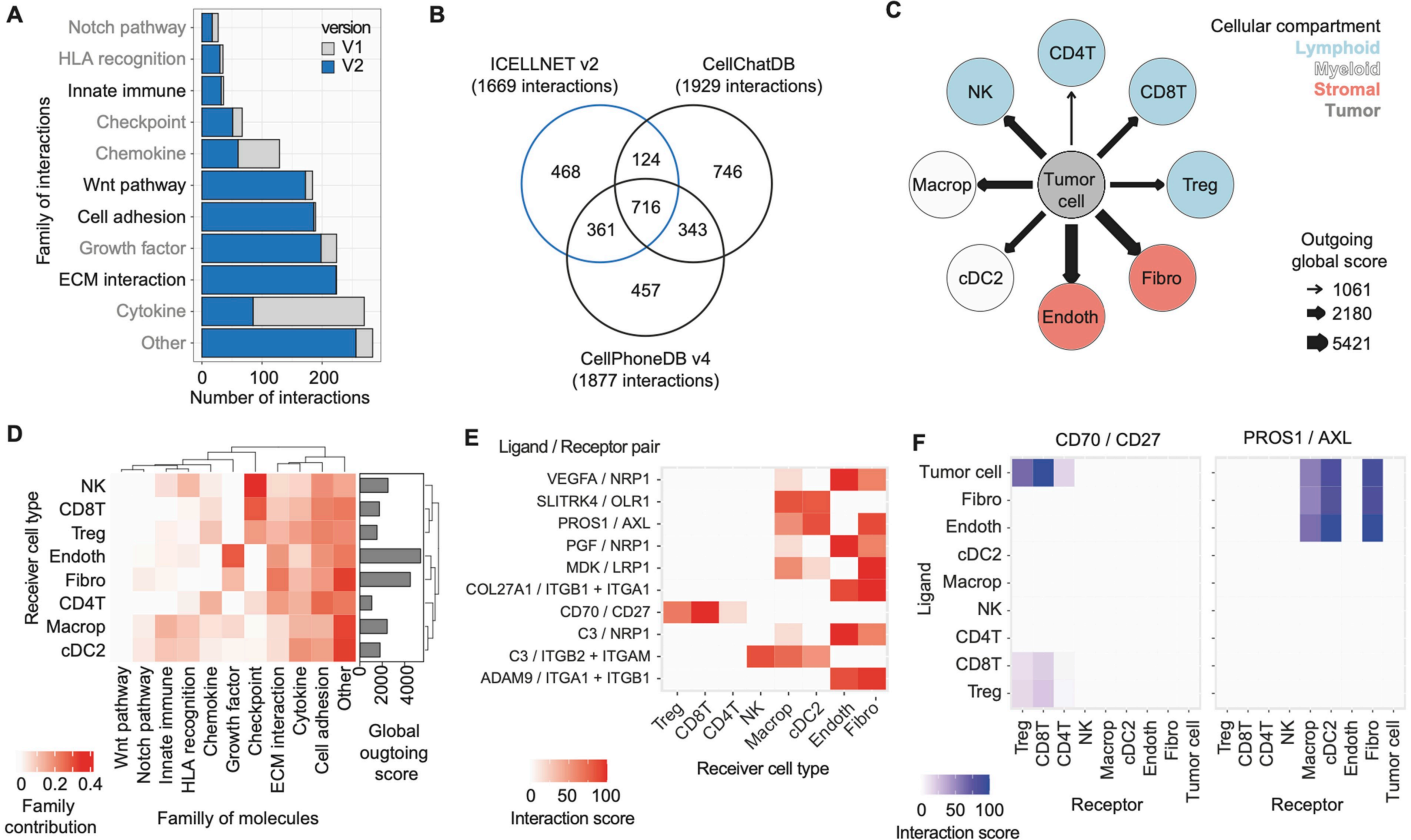
Enrichment of ICELLNET ligand–receptor database and new graphical representations (A) ICELLNET v2 interactions database, classified per family of molecules. Color code shows interactions integrated in original version (in grey) or in new version (blue). Family names included in v1 are colored in grey, whereas new families implemented in v2 are in black. ECM, extracellular matrix; HLA, human leucocyte antigen. (B) Comparison of the interactions included in ICELLNET v2, CellChat and CellPhoneDB v4 (restricted to curated protein–protein interactions) databases. Visualization in (C–F) were obtained from the analysis of scRNAseq data obtained from GSE222703. (C) Network representing the global outgoing communication scores between the emitter cells (tumor cells), and receiving cells (cell types of the tumor microenvironment). Arrow size corresponds to the intensity of the communication score. Macrop, macrophage; cDC, conventional dendritic cell; Endoth, endothelial cell; Fibro, fibroblast; Treg, regulatory T lymphocyte, T, T lymphocyte; NK, natural killer. (D) Heatmap providing the contribution (in percentage) of each family of molecules (horizontal axis) to the global outgoing communication scores (barplot on the right) between tumor cell and each receiver cell types (vertical axis), computed using ICELLNET original method and selecting only the genes expressed in at least 10% of cells per cluster. Families and cell types are grouped according to Ward-D hierarchical clustering using the Euclidean distance. (E) Heatmap showing the top 10 most variable interactions between all the conditions (pairs of tumor cell and receiver cell types), sorted according to the variance of their scores within the conditions. The heatmap displays the outgoing interaction score (from 0 to 100) between tumor cells and the receiver other cell type (horizontal axis), for each ligand/receptor interaction (vertical axis). (F) Heatmap displaying the interaction score (from 0 to 100) of *CD70/CD27* (left) and *PROS1/AXL* (right) interactions, for each pair of cell types. Emitter cells (expressing the ligand) are provided in vertical axis, and receiver cells (expressing the receptor) are provided as horizontal axis.

In its original version, ICELLNET database included 380 ligand–receptor interactions, mostly focused on cytokine-mediated interactions and immune processes. To broaden the use of ICELLNET to other biological fields and cellular systems, we expanded the ICELLNET database to a wider range of communication molecules, such as growth factors, hormones, cell adhesion molecules, molecules involved in the Wnt pathway, and neuronal signaling. For this, we manually curated additional ligand–receptor interactions experimentally demonstrated in human systems, with the help of recent ligand–receptor interaction screening studies (Voloshanenko *et al.* 2017, Wojtowicz *et al.* 2020) and the previously established resources NATMI (Hou *et al.* 2020), and CellPhoneDB (Efremova *et al.* 2020). From these resources, 199 interactions were excluded (Supplementary Data S1), mainly because they: (i) have to our knowledge not been investigated and validated in human, such as *SEMA4G/PLXNB2* (Maier *et al.* 2011), or (ii) are not occurring in humans, such as *CXCL5/CXCR1* interacting in mice only (Bachelerie *et al.* 2014). Interactions with controversial results in humans were yet included, such as *WNT3A/FZD6+LRP6* (Dijksterhuis *et al.* 2014, Voloshanenko *et al.* 2017) or *ANGPT1/ITGA5+ITGB1* (Weber *et al.* 2005; Hakanpaa *et al.* 2015). In total, 1669 interactions were integrated, involving 668 heterodimeric interactions, which corresponds to a four time increase of the database content as compared to its original version (Fig. 1A and Supplementary Data S2). Similarly, we extended the classification of the interactions into 10 main families, based on molecules involved. As compared to its initial version, the previously existing families “Checkpoint,” “Growth factor,” and “HLA recognition” (previously named “Antigen binding”) were greatly expanded, and four additional families were created: “Wnt pathway,” “Innate immune,” “Cell adhesion,” and interactions involving components of the extracellular matrix (ECM) “ECM interaction” (Fig. 1A).

Comparing ICELLNET with two other curated databases considering heterodimers, CellChat and CellPhoneDB, showed around 72% overlap between ICELLNET and at least one other resource, but also 468 interactions uniquely described in ICELLNET (Fig. 1B). A previous study (Dimitrov *et al.* 2022) has shown that existing ligand–receptor databases tend to account for different interactions between the same proteins, which drastically impacts cell–cell communication analyses. Divergence in database content may come from differences in the curation strategy (inclusion of previous resources versus manual curation), the level of experimental evidence required, or other more specific criteria such as restriction of knowledge to specific species. In ICELLNET, only interactions experimentally demonstrated in human systems were considered. Thus, around 50% of interactions not included in ICELLNET but shared by CellChat and CellPhoneDB corresponded to interactions from Wnt pathway, where biological knowledge largely derives from non-human studies (Rim *et al.* 2022).

Finally, compared to other resources, the structure of ICELLNET database is simple and intuitive, gathering ligand and receptor genes information, family classification, and literature references in a single table. The database can thus be easily exploited by non-experts in the field, while the CellPhoneDB and CellChat databases have more complex structures divided into several complementary files.

## 3 Methodological implementations

### 3.1 Optimization of ICELLNET for scRNAseq data analysis

ICELLNET was originally developed to infer cell–cell communication from transcriptomic profiles of sorted cell types using bulk RNAseq or microarrays, or averaged scRNAseq data. Starting from the normalized gene expression matrix, ICELLNET first scales expression levels from 0 to 10 per gene to give similar weight to each gene and not favor highly expressed genes for the computation of communication scores. However, when considering scRNAseq data, this step can introduce false positive expression for genes that are not expressed frequently enough. Those can correspond to noise or low expressed genes suffering from dropout. Thus, to decrease the number of false positive results, we added a filtering step to keep only the genes detected in a sufficient proportion of cells, generally in more than 5%–10% of cells in a cluster (threshold fixed by the user), depending on the objective of the analysis. This step is not applicable to other transcriptomic type of data (RNAseq, microarray, spatial transcriptomic). The communication scores between cells are then computed as previously described (Noël *et al.* 2021).

### 3.2 New visualizations to help prioritization and biological interpretation

Increasing the number of considered ligand–receptor interactions in ICELLNET analyses ultimately complexify the results and the biological interpretation. Thus, appropriate visualizations are needed to identify communication channels of interest, prioritize, and interpret the results. One strength of ICELLNET is that it offers several representations at different layers, displaying: (i) global communication scores between cells as a network (Fig. 1C); (ii) global contribution of each family of molecules to the communication score; (iii) interaction scores for specific ligand–receptor pairs of interest as dot plot or heatmap. In this new version, we adapted the graphical representation of the contribution of families of molecules (Fig. 1D) and applied it to a scRNAseq dataset containing cells from human renal carcinoma tumor microenvironment (GSE222703). Previously represented as a stacked bar plot, the relative contribution of each family of molecules to the communication score was updated to a heatmap to better reflect the new database nomenclature and to improve readability of the results. Thus, in Fig. 1D, tumor cells were shown to mainly interact with endothelial cells using growth factors, while the largest contributions of checkpoint interactions were observed on tumor cell interactions with CD8 T lymphocytes (CD8 T) and NK (natural killer) cells.

In addition, we implemented new graphical representations to facilitate prioritization of the results, for example by easily displaying the n (number provided by the user) most contributing interactions or most variable interactions between all the conditions, i.e. cell types (Fig. 1E). Most variable interactions are sorted by the score variance across the different conditions. Thus, in Fig. 1E, tumor cells used only one interaction among the top 10, *CD70/CD27* interaction, to communicate with CD8 T and regulatory T cell, while five interactions were mostly used to interact with both endothelial cells and fibroblasts. The four remaining interactions were mainly dedicated to interacting with macrophages, dendritic cells (cDC2) or NK cells. Similarly, we added the possibility to visualize and compute specific ligand–receptor interaction scores for all possible cell pairs. Thus, in Fig. 1F, *CD70/CD27* interaction was almost exclusively used by tumor cells to interact with lymphocytes whereas *PROS1/AXL* interaction was shared by tumor cells, endothelial cells, and fibroblasts to interact with macrophages, cDC2, and fibroblasts. This feature can be very helpful to elaborate prioritization strategies, such as selecting interactions exclusively used by one cell type in a dataset (Massenet-Regad *et al.* 2023). Further details on the use of these representations can be found in the package documentation.

## 4 Conclusions

ICELLNET is a versatile method that can be applied to a wide range of transcriptomic data, including bulk RNAseq, scRNAseq, and spatial transcriptomics. The major enrichment of the ICELLNET ligand–receptor database to cover more biological areas will extend its performance and broaden its application fields. New graphical visualizations are crucial to facilitate results prioritization and interpretation and will provide novel biological insights.

## Supplementary Material

btae089_Supplementary_Data

## Data Availability

The processed data used in this study are available under the accession number GSE222703, and the code used to generate the figures is accessible at Github (https://github.com/lmassenet-regad/Paper_ICELLNET_V2).
